# Arthroscopic Management and Radiographic Interpretation of an Everted Bony Bankart Lesion

**DOI:** 10.1155/2018/9261260

**Published:** 2018-05-29

**Authors:** Alexander J. Hron, Benjamin C. Noonan

**Affiliations:** ^1^University of North Dakota, 1301 N. Columbia Rd., Grand Forks, ND 58203, USA; ^2^Sanford Health, Sanford Orthopedics & Sports Medicine, 2301 25th Street S., Fargo, ND 58103, USA

## Abstract

Soft tissue injuries are prevalent after traumatic anterior shoulder dislocation. However, bony fractures, often referred to as bony Bankart injuries, are less common. The authors describe the case of a 16-year-old male who displayed a bony Bankart with a unique, everted presentation. The patient presented with left shoulder pain, restricted range of motion, and crepitus. Two weeks prior to physical examination, he sustained a traumatic anterior glenohumeral dislocation after a bicycle accident, which reduced spontaneously. Plain film imaging revealed a bony fragment off the anterior glenoid. Upon critical examination of magnetic resonance imaging axial cuts, the bony fragment was found to be flipped. Intraoperatively, this orientation was confirmed. The fragment was reduced and stabilized in an anatomic position using a double row technique with the capsule then advanced over the top of the fragment using three additional anchors. Imaging four months postoperatively revealed an anatomical reduction of the fragment. To the authors' knowledge, this is the first reported case of bony fragment eversion following traumatic anterior shoulder dislocation. Although the incidence of everted bony fragments following traumatic dislocation is unknown, such a situation presents unique challenges to the orthopedic surgeon. The authors discuss potential eversion mechanisms, fragment identification by imaging, surgical indications, and operative techniques.

## 1. Introduction

The shoulder is the most mobile joint in the body, allowing for a large range of motion (ROM). The humeral head relies on the glenoid, along with a passive and dynamic system of ligaments and muscles to resist dislocation. In traumatic shoulder instability, this system is disrupted, which can result in displacement of the labrum from the glenoid and the formation of a rim fracture known as a bony Bankart. The incidence of traditional soft tissue Bankart lesions associated with traumatic anterior shoulder dislocation has been reported to be as high as 90 percent; however, a bony Bankart occurs in only a small percentage of these cases [1]. Additionally, a bony Bankart with eversion is presumed to be much less common, with this being the first described case to the authors' knowledge. The authors discuss potential eversion mechanisms, imaging, surgical indications, and operative techniques.

## 2. Case

A 16-year-old male presented for evaluation of left shoulder pain, crepitus, and reduced ROM. Two weeks prior, he sustained a traumatic glenohumeral dislocation after a bicycle accident, which reduced spontaneously. Early rehabilitation provided no improvement to his restricted ROM or pain.

**Figure 1:**
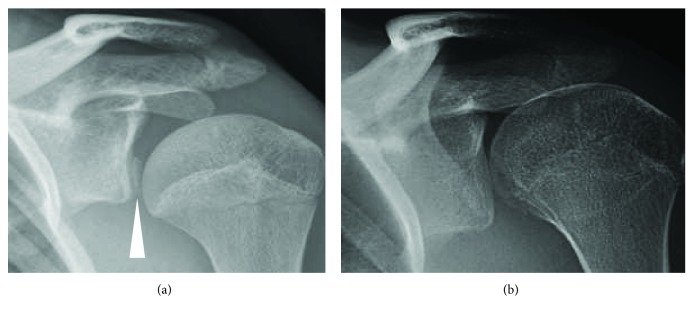
Anteroposterior plain film radiographs of the patient's left shoulder. Bony fragment off the anterior glenoid preoperative indicated by arrow (a). Successful anatomical reduction of the bony fragment four months postoperative (b).

**Figure 2:**
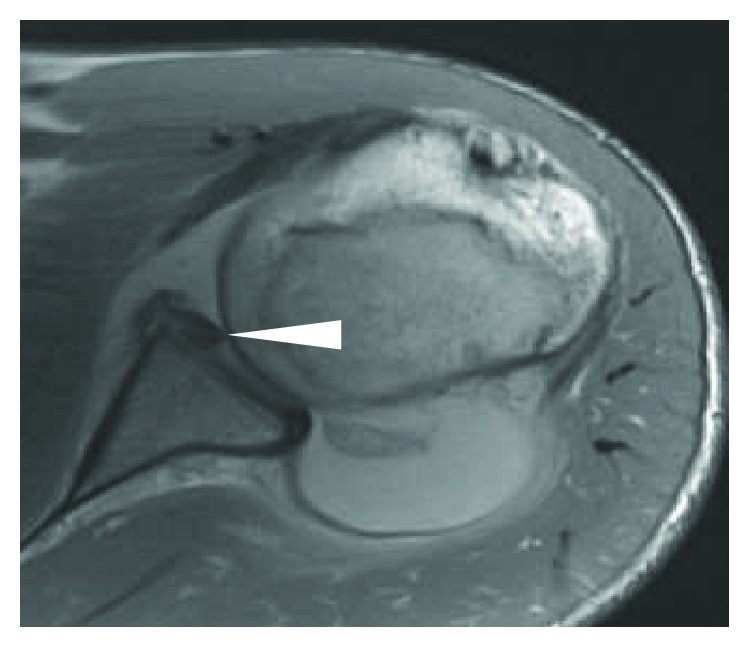
Axial T1 weighted MRI shoulder slice of the left shoulder with an arrow pointing to the everted fragment.

On physical examination, the patient appeared uncomfortable and extremely guarded. Apprehension and load and shift testing were consistent with anterior shoulder instability, and his ROM seemed reduced compared to typical dislocations. Plain film radiography revealed a 1 cm thin fragment of bone off the anterior glenoid, indicative of a bony Bankart (Figure 1(a)). Magnetic resonance imaging (MRI) confirmed this, but on critical examination of axial cuts, the fragment appeared flipped with subchondral bone superficial to articular cartilage (Figure 2). Based on his pain and the redislocation rate of his age group after traumatic dislocations, surgery was recommended [2].

**Figure 3:**
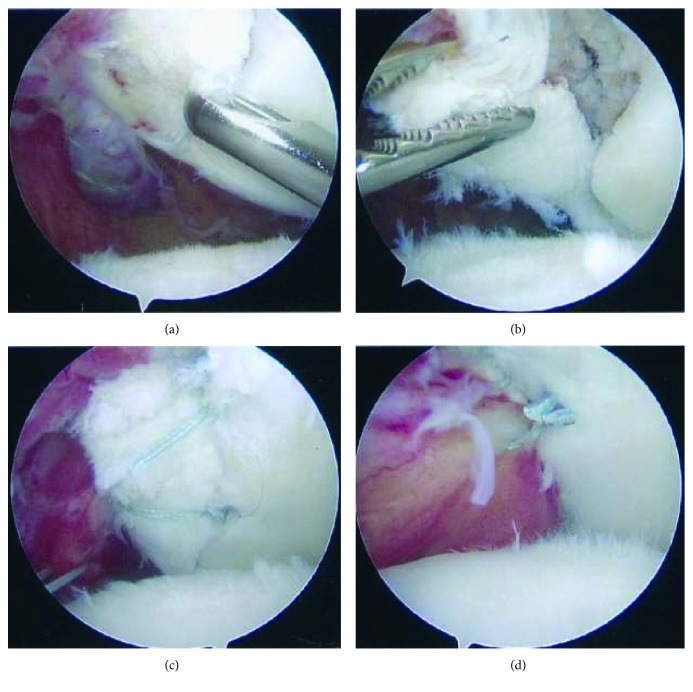
Intraoperative images of the left shoulder from the anterior high rotator interval portal. Bony fragment with subchondral bone superficial and articular cartilage deep (a). Reduction of fragment with grasper (b). Fragment anatomically reduced and fixed in place with suture anchors (c). Capsular layer repaired over the top of the fragment and fixed to intact glenoid utilizing knotless anchors (d). Humeral head abrasion is apparent in all images.

**Figure 4:**
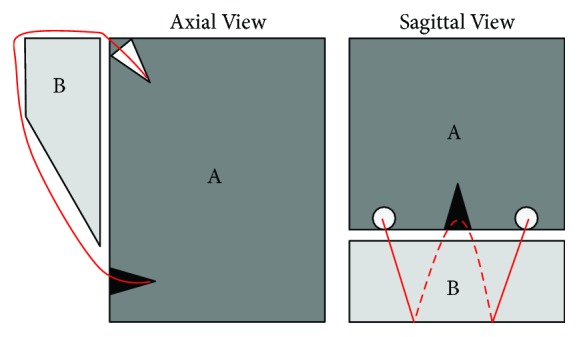
Sagittal and axial view of glenoid (A) and fragment (B). Two anchors were placed near the articular surface of the glenoid (depicted in white), and one was placed in the scapula below the fragment (depicted in black). The fragment was fixed in place by connecting the suture (depicted in red) from both anchors close to the articular surface of the glenoid to the anchor below the fragment.

Intraoperatively, a posterior portal was created using bony landmarks, after which anterior and high rotator interval portals were established with the assistance of spinal needle localization to achieve an appropriate trajectory onto the glenoid. A 30° arthroscope was placed in the high interval portal, adjacent to the biceps tendon, and was used for subsequent exploration of the joint and image capture. A thin bony fragment with the articular surface deep and the bony surface superficial was present (Figures 3(a) and 3(b)). Both articular cartilage abrasion (Figure 3), possibly from articulating with the exposed subchondral fragment, and a small Hill-Sachs lesion were present on the humeral head. The anterior portal was used to allow access for a liberator, grasper, and rasp which were used to free up the partially healed fragment, achieve reduction, and freshen bony edges. A transsubscapularis (TSS) portal, as described by Dwyer et al., was established using a percutaneous set (Arthrex, United States) and a small metal 5.4 mm cannula to minimize potential injury to the subscapularis muscle [3]. The fragment was fixed using a double row technique as described by Millet and Braun using three Arthrex PushLock 2.9 mm anchors (Arthrex, United States) and #2 FiberWire suture (Arthrex, United States) (Figures 3(c) and 4) [4]. The capsule was then advanced over the top of the fragment using three additional PushLock anchors (Figure 3(d)).

Postoperatively, the patient had no major complications. He followed a graduated-intensity 18-week institutional physical therapy program consisting of two three-week and two six-week phases, which included passive and active ROM exercises and strengthening of shoulder musculature (Table 1). Plain film imaging four months postoperative indicated anatomic reduction of the fragment (Figure 1(b)). On examination, active shoulder ROM was assessed for both the repaired shoulder and the contralateral shoulder. Compared with the contralateral shoulder, the repaired shoulder exhibited the same ROM but lacked 15° of external rotation with hands at sides and 15° with the arm at 90° abduction. The patient was seen again six months postoperative and on examination exhibited excellent strength and no instability symptoms but still lacked 15° of external rotation both with arms at the sides and at 90° of shoulder abduction. By this point in time, the patient had resumed sporting activities including cross-country skiing and track and field.

## 3. Discussion

Although injury mechanisms for traditional bony Bankart lesions are known, mechanisms that produce an everted bony fragment have not yet been elucidated. A traumatic anterior shoulder dislocation is often incited by a fall on an outstretched hand, with the arm in external rotation and abduction [1]. In this position, the inferior glenohumeral ligament (IGHL) is the primary restraint to dislocation [1]. A bony Bankart injury results when an avulsion fracture occurs at the insertion of the IGHL [1]. Eversion may result after creation and partial displacement of the bony fragment, possibly due to cartilage of the humeral head catching on the edge of the articular surface of the bony fragment, pulling it deep. A similar situation could result during relocation. An important consideration in determining the possibility of an everted fragment is the anatomy of the fragment. At one centimeter, this fragment was relatively superficial, almost like a “wafer,” and was unlike injuries in which the fragment includes the deeper bone, which likely would have prevented eversion.

Some clues were present during the physical examination that led us to consider the possibility of atypical pathology, such as severe discomfort and guarding in excess of what is normally presented with traditional traumatic shoulder dislocations, perhaps due to the articulation of the humeral head articular cartilage with the exposed subchondral bone or the mobility of the thin fragment. In conjunction with physical examination findings, preoperative imaging is important in surgical and rehabilitative planning for traumatic anterior shoulder dislocations. Both plain films and MRI revealed a thin fragment of bone off the anterior glenoid, and MRI revealed further information that it was everted based on bone signal intensity with articular bone deep and subchondral bone superficial. While the everted fragment would have eventually been covered with capsular tissue if it were left malreduced, this may have led to suboptimal healing with the articular cartilage lying adjacent to the donor site bone.

A surgical plan was established by determining the fragment width relative to the diameter of the glenoid on imaging [5, 6]. Generally, less than 12.5% bone loss will be amenable to arthroscopic restoration of capsulolabral soft tissue tension without fragment excision. In patients that have between 12.5% and 25% bone loss, fragment reduction and fixation is recommended. Alternative techniques using bone grafts, such as the Latarjet and Bristow procedures, are generally reserved for bone loss exceeding 20–30% [7, 8]. We felt that in our case, with a fragment that was over 1 cm long and approximately 20% of the anterior-posterior width of the glenoid, surgical stabilization was the best treatment option.

To achieve appropriate surgical stabilization, the TSS approach was chosen to provide the best chance for optimal fixation of the fragment. Both the low anterior (LA) approach and the TSS approach are commonly used in the repair of bony Bankart injuries. However, the LA approach has the drawback of a high angle of insertion in the coronal plane when placing an anchor near the inferior glenoid rim. Dwyer et al. investigated the TSS approach and evaluated it alongside the LA approach in cadaveric models and found that it improved the angle of approach to the inferior glenoid, potentially reducing the chances of poor anchor fixation or skiving during placement [3]. Additionally, along with bone cortical thickness and design of the anchor, the position of the anchor within the bone is critical in resisting pullout over time with repetitive motion [9]. Drawbacks of the TSS approach include greater risk to important neurovascular structures such as the musculocutaneous nerve, the axillary nerve and artery, the cephalic vein, and the subscapularis tendon; however, to our knowledge, these injuries have not been documented clinically [10]. Additionally, the use of a very small cannula inserted through a 3 mm percutaneous incision reduces the risk of damage. After considering the risks and benefits of each approach and the need for access to the more medial glenoid neck, the TSS approach was chosen with a careful eye to detail and anatomical landmarks to minimize the possibility of damage to neurovascular structures.

Following surgery, the patient was enrolled in a graduated-intensity 18-week institutional physical therapy program, through which he progressed satisfactorily. Clinical examination of the patient at four and six months postoperative, however, did reveal a 15° ROM deficit in external rotation at the repaired shoulder. Loss of external rotation has been reported as the most common limitation on motion following instability surgery, with one retrospective study finding a prevalence of 8% and another finding a prevalence of 16.1% [11, 12]. However, the loss of external rotation in this situation was minimal, and the patient had an excellent functional outcome evidenced by his subsequent return to sports activity.

Some physical examination findings, such as excessive guarding, may point towards the presence of atypical pathology and in rare cases an everted fragment. MRI should be used to determine the size and orientation of the fragment, after which a surgical approach, based on glenoid bone loss, should be devised. In this case, an everted fragment was identified on MRI through identification of labrum medialization. MRI was also used to determine glenoid bone loss, which was calculated to be 20%. Resultantly, a modified fragment reduction and fixation procedure using an arthroscopic TSS approach was determined as the appropriate choice and ultimately resulted in a desirable outcome.

## Figures and Tables

**Table 1:** 

*Weeks 1–3: phase I*	

Sling	At all times (including sleeping) when not doing exercises

Exercises	Passive forward flexion (FF) in scapular plane to 90°
	Passive external rotation (ER) and extension to neutral
	Elbow/wrist active ROM
	Scapular isometrics
	Pain-free submaximal deltoid isometrics
	Modalities as needed

Advancement criteria	ER to neutral/FF to 90°/minimal pain or inflammation

*Weeks 3–6: phase II*	

Sling	At all times when not doing exercises

Exercises	Active assisted FF in scapular plane to 120°: wand exercises, pulleys
	Active assisted ER to 30°: wand exercises
	Manual scapula side-lying exercises
	Internal/external rotation isometrics in modified neutral (submaximal, pain-free)
	Modalities as needed

Advancement criteria	Minimal pain and inflammation
	ER to 45°/FF to 120°
	IR/ER strength 4/5

*Weeks 6–12: phase III*	

Sling	May discontinue

Exercises	Active assisted FF in scapular plane to tolerance
	Active assisted ER to tolerance (go slow with ER)
	Begin active assisted ROM for internal rotation
	Progress scapular strengthening—include closed chain exercises
	Begin isotonic IR/ER strengthening in modified neutral (pain-free)
	Begin latissimus strengthening (progress as tolerated)
	Begin humeral head stabilization exercises (if adequate strength and ROM)
	Begin upper extremity flexibility exercises
	Isokinetic training and testing
	Modalities as needed

Advancement criteria	Normal scapulohumeral rhythm
	Minimal pain and inflammation
	IR/ER strength 5/5
	Full upper extremity ROM
	Isokinetic IR strength 85% of unaffected side

*Weeks 12–18: phase IV*	

Exercises	Progress to full functional ROM
	Advance IR/ER strengthening to 90/90 position if required
	Continue full upper extremity strengthening program
	Continue upper extremity flexibility exercises
	Isokinetic strengthening and testing
	Activity-specific plyometrics program
	Address trunk and lower extremity demands
	Begin sport or activity-related program

Discharge criteria	Pain-free sport or activity-specific program
	Isokinetic IR/ER strength equal to unaffected side
	Independent home exercise program
